# Too Much Heat May Make You Smoke

**DOI:** 10.31586/gjcd.2025.1175

**Published:** 2025-01-15

**Authors:** Shervin Assari, Babak Najand, Hossein Zare

**Affiliations:** 1 Department of Internal Medicine, Charles R. Drew University of Medicine and Science, Los Angeles, CA, United States; 2 Department of Urban Public Health, Charles R. Drew University of Medicine and Science, Los Angeles, CA, United States; 3 Marginalization-Related Diminished Returns (MDRs) Center, Los Angeles, CA, United States; 4 Department of Health Policy and Management, Johns Hopkins Bloomberg School of Public Health, Baltimore, MD, United States; 5 School of Business, University of Maryland Global Campus (UMGC), Adelphi, MD, United States

**Keywords:** Extreme Heat, Climate Change, Substance Use, Tobacco Use, Child Development, Socioeconomic Status, Vulnerable Populations

## Abstract

**Background:**

The rising concerns surrounding climate change have drawn attention to its potential impact on health, particularly among vulnerable groups such as children and older adults. Despite extensive research on health consequences, limited studies have explored the connection between extreme heat exposure and tobacco use initiation among adolescents in the United States.

**Objectives:**

This study examines the relationship between extreme heat exposure and the initiation of tobacco use in adolescents, using data from the Adolescent Brain Cognitive Development (ABCD) study. It also evaluates the mediating roles of major depressive disorder (MDD) and childhood behavioral problems.

**Methods:**

Data from 11,878 participants in the ABCD study were analyzed to explore the link between extreme heat exposure (independent variable) and tobacco use initiation (dependent variable). Covariates included age, sex, and race/ethnicity, while MDD and behavioral problems (measured using the Child Behavior Checklist, CBCL) were assessed as potential mediators. Structural equation modeling (SEM) was applied for analysis.

**Results:**

The findings indicated a significant association between extreme heat exposure and an increased likelihood of tobacco use initiation in adolescents aged 9 to 15. MDD and behavioral problems partially mediated this relationship.

**Conclusions:**

These results underscore the importance of targeted interventions aimed at mitigating the impact of extreme heat on adolescent health, including its influence on tobacco use initiation. Addressing mental health and behavioral challenges could help reduce these risks. Future longitudinal research is needed to confirm these findings and evaluate the efficacy of strategies to protect vulnerable youth populations.

## 1. Introduction

Global temperatures have reached unprecedented levels over the past decade, marking the most significant climatic shifts since the mid-19th century. The Intergovernmental Panel on Climate Change (IPCC) [1] predicts that climate change will continue to drive an increase in the frequency and intensity of extreme heat events. These trends raise significant concerns about the wide-ranging effects of extreme weather, particularly in economically disadvantaged regions, where communities often lack resources to mitigate environmental stressors [2]. Economically, extreme weather events can disrupt local economies by increasing operational costs, reducing labor productivity, diminishing agricultural yields, and negatively impacting both consumer demand and business operations [3–6].

The health implications of extreme heat are equally concerning [7, 8]. Heat exposure has been linked to elevated mortality and morbidity rates, adverse pregnancy outcomes, and worsening mental health. Heat stress can impair physical and cognitive performance, exacerbate occupational health risks, and reduce productivity [2]. Globally, nearly half the population, including over 1 billion workers, experiences high heat episodes, with a significant proportion suffering negative health effects [2]. Despite the severity of these outcomes, many heat-related health risks can be mitigated through action plans incorporating behavioral and technological solutions [2]. Urban areas face compounding risks due to heat generated by vehicular traffic and building emissions [2].

Children are particularly susceptible to the adverse effects of extreme heat. Studies indicate that younger children (ages 0–4) are at an increased risk of emergency department visits on days with higher maximum temperatures, with same-day exposure showing the strongest correlations. For example, an increase in maximum temperature (Tmax) by 13°F is associated with a 2.6% increase in emergency visits (95% CI: 2.2–3.0). Similar patterns have been observed across racial/ethnic groups and for a variety of health outcomes, including heat-related illnesses, injuries, and infectious diseases [9, 10].

The Centers for Disease Control and Prevention (CDC) emphasizes that children and adolescents, especially those in poverty, are particularly vulnerable to heat exposure. For these groups, extreme heat may disrupt daily routines, including play and social interactions, leading to isolation and diminished community engagement. Children in low-income households often lack access to mitigating resources such as air conditioning, which exacerbates their vulnerability. Despite these risks, research on the developmental and behavioral impacts of extreme heat exposure in youth remains limited.

Extreme heat exposure may influence youth behavior, including high-risk behaviors such as tobacco use initiation. While studies have established the physical health consequences of heat waves, there is limited exploration of their behavioral and psychosocial effects. Evidence suggests that neighborhood socioeconomic status (SES), financial hardship, peer influences, and puberty-related changes may mediate the relationship between environmental stressors and youth behaviors [26–29]. In a recent study that used data from the Adolescent Brain Cognitive Development (ABCD) study, Assari and Zare explored the association between extreme heat exposure and delinquency among 11,878 children, examining potential mediators including neighborhood socioeconomic status (SES), puberty, peer deviance, and financial difficulties. Findings revealed that children exposed to higher levels of extreme heat were more likely to reside in low-SES neighborhoods, face financial hardships, experience advanced puberty, and engage in higher levels of delinquent behavior. These results underscore the disproportionate impact of extreme heat on socioeconomically disadvantaged youth, highlighting the urgent need for targeted interventions to mitigate these effects and address underlying inequities. Future research should adopt longitudinal approaches to evaluate effective strategies for protecting vulnerable populations [48].

### Objectives:

This paper seeks to address this research gap by investigating the relationship between extreme heat exposure and tobacco use initiation among adolescents. Using data from the Adolescent Brain Cognitive Development (ABCD) study [16–25], we examined how environmental stressors influence youth behaviors, with a particular focus on tobacco use. Additionally, we explored the potential mediating roles of major depressive disorder (MDD) and childhood behavioral problems. By advancing understanding in this area, this research aims to inform the development of effective mitigation and intervention strategies to protect youth from the adverse impacts of climate change and extreme heat.

## 2. Methods

### 2.1. Design and Sample

This study utilized secondary data from the Adolescent Brain Cognitive Development (ABCD) study [16–25], a large-scale, longitudinal research project involving a diverse sample of pre-adolescent children from various racial, ethnic, and socioeconomic backgrounds. The methodology for the ABCD study has been extensively detailed in previous publications. Key strengths of this dataset include its national coverage, longitudinal design, and diversity across race, socioeconomic status (SES), and geographical regions. Participants were primarily recruited through schools.

### 2.2. Analytical Sample

The analytical sample included all eligible youth from the ABCD study, irrespective of their racial, ethnic, or economic background. Participants were 9–10 years old at the baseline assessment, and a total of 11,878 children were included in this analysis.

### 2.3. Ethics

This research was reviewed and approved by the Institutional Review Board (IRB) at the University of California, San Diego (UCSD). Written informed consent was obtained from parents, while children provided their assent to participate.

### 2.4. Study Variables

#### Race/Ethnicity:

Parent-reported data on the race and ethnicity of participants were categorized into non-Latino White (reference group), Black, Latino, Asian, and other racial/ethnic groups.

#### Family SES:

Socioeconomic status was assessed using indicators such as household income and parental education levels.

#### Childhood Behavioral Problems:

Behavioral issues were measured using the Child Behavior Checklist (CBCL), where higher scores indicated greater behavioral challenges.

#### Major Depressive Disorder (MDD):

MDD was assessed using the K-SADS diagnostic interview, which collects data from parents about psychiatric symptoms in children. This variable was coded as a binary indicator reflecting the presence or absence of MDD.

#### Tobacco Use Initiation:

Tobacco use initiation was defined as the first instance of reported use of any tobacco product, including cigarettes, e-cigarettes, and other related products, during any follow-up period.

#### Neighborhood Median Home Value:

This variable was derived from zip code-level data in the ABCD study’s residential history, representing the median home value in the neighborhood as a continuous measure of area-level SES.

### 2.5. Data Analysis

Data were analyzed using Stata software. Univariate analyses summarized continuous variables using means and standard deviations (SD). Bivariate relationships were assessed through Pearson correlation tests. Multivariable analyses employed structural equation modeling (SEM) to examine pathways linking state-level heat wave exposure to tobacco use initiation. Mediators included childhood behavioral problems and MDD. Covariates such as age, sex, race/ethnicity, family SES, and neighborhood SES were included as potential confounders. Multicollinearity was checked and ruled out (all correlations were below 0.6). Results were reported as standardized path coefficients (beta), 95% confidence intervals (CI), and p-values.

## 3. Results

### Association Between Extreme Heat Exposure and Mediators

The structural equation model (SEM) revealed a significant positive association between heat exposure and major depressive disorder (MDD) (B = 0.032, SE = 0.011, 95% CI: 0.011–0.054, p = 0.003) as well as heat exposure and behavioral problems (B = 0.030, SE = 0.010, 95% CI: 0.011–0.049, p = 0.002). These findings suggest that exposure to extreme heat is associated with worse mental health and higher levels of behavioral problems in children.

### Mediators and Tobacco Use Initiation

MDD was positively associated with tobacco use initiation (B = 0.024, SE = 0.011, 95% CI: 0.003–0.046, p = 0.027), as were behavioral problems (B = 0.058, SE = 0.009, 95% CI: 0.040–0.077, p < 0.001). These results indicate that both MDD and behavioral problems play significant roles in mediating the relationship between heat exposure and tobacco use initiation.

### Direct Effects on Tobacco Use Initiation

Heat exposure had a direct positive association with tobacco use initiation (B = 0.046, SE = 0.010, 95% CI: 0.027–0.065, p < 0.001). Additional predictors of tobacco use initiation included age (B = 0.076, SE = 0.009, 95% CI: 0.058–0.094, p < 0.001), male gender (B = −0.021, SE = 0.009, 95% CI: −0.039 to −0.003, p = 0.024), family income (B = −0.033, SE = 0.014, 95% CI: −0.061 to −0.006, p = 0.017), and neighborhood income (B = 0.021, SE = 0.011, 95% CI: 0.000–0.043, p = 0.049).

### Sociodemographic Predictors

Black children were less likely to initiate tobacco use compared to non-Latino White children (B = −0.030, SE = 0.011, 95% CI: −0.052 to −0.008, p = 0.007), whereas Asian children also showed lower tobacco use initiation (B = −0.027, SE = 0.009, 95% CI: −0.045 to −0.008, p = 0.004). Latino ethnicity and other racial/ethnic groups did not show significant differences in tobacco use initiation.

### Behavioral Problems

Behavioral problems were significantly associated with several sociodemographic factors. Male gender was positively associated with higher levels of behavioral problems (B = 0.108, SE = 0.009, 95% CI: 0.090–0.125, p < 0.001), while family income showed a negative association (B = −0.190, SE = 0.013, 95% CI: −0.216 to −0.163, p < 0.001). Heat exposure was also positively associated with behavioral problems (B = 0.030, SE = 0.010, 95% CI: 0.011–0.049, p = 0.002).

## 4. Discussion

The primary objective of this study was to examine the relationship between extreme heat exposure and tobacco use initiation among adolescents, using data from the Adolescent Brain Cognitive Development (ABCD) study [16–25]. In addition, the study aimed to explore socio-demographic factors such as race/ethnicity, family and neighborhood SES, and financial difficulties that may heighten vulnerability to extreme heat exposure. Mediators, including major depressive disorder (MDD) and childhood behavioral problems, were investigated to better understand the pathways through which heat exposure influences tobacco use initiation.

Our findings revealed a significant association between extreme heat exposure and tobacco use initiation. Adolescents exposed to higher levels of heat were more likely to reside in low-SES neighborhoods, come from financially disadvantaged families, and report higher levels of behavioral problems and MDD. These results underscore the compounding effects of socio-environmental stressors, such as extreme heat, on vulnerable populations, particularly youth already experiencing socioeconomic disadvantages.

The disproportionate exposure to extreme heat among Black youth can be attributed to several structural and historical factors. Historical legacies of systemic racism and segregation have concentrated Black populations in regions prone to extreme heat, such as the southern United States, which are also marked by higher poverty rates [30–34]. Black communities are more likely to live in urban areas with limited green spaces, poor infrastructure, and environmental conditions that exacerbate the urban heat island effect [35–37]. These factors amplify the physiological and psychological stress associated with extreme heat, increasing the likelihood of adverse health behaviors such as tobacco use initiation.

Youth from lower SES families are similarly disadvantaged, as they are less likely to have access to adequate cooling systems and live in housing with proper insulation [38–42]. Financial hardships compound these risks by limiting families’ ability to invest in cooling solutions or relocate to cooler environments, leaving children more vulnerable to heat-related stress. This chronic stress can contribute to behavioral and emotional regulation challenges, increasing the likelihood of tobacco use as a coping mechanism.

Neighborhood-level socioeconomic disadvantage further amplifies these risks. Communities with lower SES often lack public cooling resources, such as parks, pools, or air-conditioned facilities, and are frequently situated in areas with poor infrastructure and higher pollution levels [44]. These conditions create a cumulative stress burden, increasing susceptibility to behavioral health issues, including tobacco use initiation.

The mediating roles of MDD and childhood behavioral problems offer additional insight into how extreme heat exposure translates into tobacco use initiation. Physiological stress caused by extreme heat can exacerbate emotional dysregulation and irritability, contributing to both mental health challenges and risky health behaviors. Adolescents with preexisting behavioral problems or depressive symptoms may be particularly vulnerable, as these conditions can impair decision-making and increase susceptibility to peer influence, further heightening the risk of initiating tobacco use.

### 4.1. Implications

These findings have critical implications for public health and social policy. Targeted interventions are essential to protect vulnerable populations from the adverse effects of extreme heat. Strategies should include improving housing quality, ensuring access to affordable cooling systems, and establishing community cooling centers in disadvantaged neighborhoods. Public health campaigns should also address the mental health and behavioral risks associated with extreme heat exposure, equipping families with tools to reduce their children’s vulnerability. Schools and community organizations may play a pivotal role by providing safe, air-conditioned spaces and organizing structured activities during heatwaves.

### 4.2. Future Research

Future studies should focus on longitudinal designs to track the long-term impact of extreme heat exposure on tobacco use and other health behaviors. Understanding the cumulative effects of repeated heat exposure across developmental stages will provide insights into chronic stress mechanisms and their behavioral consequences. Research should also explore the intersection of extreme heat with other environmental stressors, such as air pollution and food insecurity, to develop a comprehensive framework for addressing multiple layers of disadvantage.

Additionally, there is a need to evaluate the effectiveness of mitigation strategies such as urban greening, energy-efficient housing, and cooling centers in reducing the health and behavioral impacts of extreme heat. Exploring how schools and public health campaigns can effectively educate families about heat safety practices will also be critical. Tailored interventions that account for regional and demographic variations in vulnerability will ensure that resources are equitably distributed to those most at risk.

### 4.3. Limitations

This study has several limitations. First, tobacco use initiation was self-reported, which may be subject to social desirability and recall biases. Future studies should incorporate objective measures, such as biomarkers or clinical records, to validate self-reported data. Second, the cross-sectional design limits the ability to establish causation or temporal relationships between heat exposure, mediators, and outcomes. Longitudinal research is necessary to better understand the directionality of these associations.

Additionally, other potential confounding factors, such as parental supervision and access to recreational facilities, were not fully accounted for in this analysis. Including these variables in future research could enhance the robustness of findings. Finally, while the ABCD study provides a racially and economically diverse sample, the results may not be generalizable to all geographic regions or demographic groups. Despite these limitations, the study offers valuable insights into the behavioral health impacts of extreme heat exposure and highlights the need for targeted interventions.

## 5. Conclusion

This study demonstrates a significant association between extreme heat exposure and increased risk of tobacco use initiation among adolescents, with MDD and behavioral problems serving as important mediators. Vulnerable populations, including Black youth and those from lower SES families and neighborhoods, face compounded risks due to socio-environmental disparities. The findings underscore the urgent need for targeted policies and interventions to mitigate the effects of extreme heat and address the underlying inequities that exacerbate these risks. As climate change continues to intensify, protecting vulnerable populations is critical to ensuring the health and well-being of future generations.

## Figures and Tables

**Figure 1.**
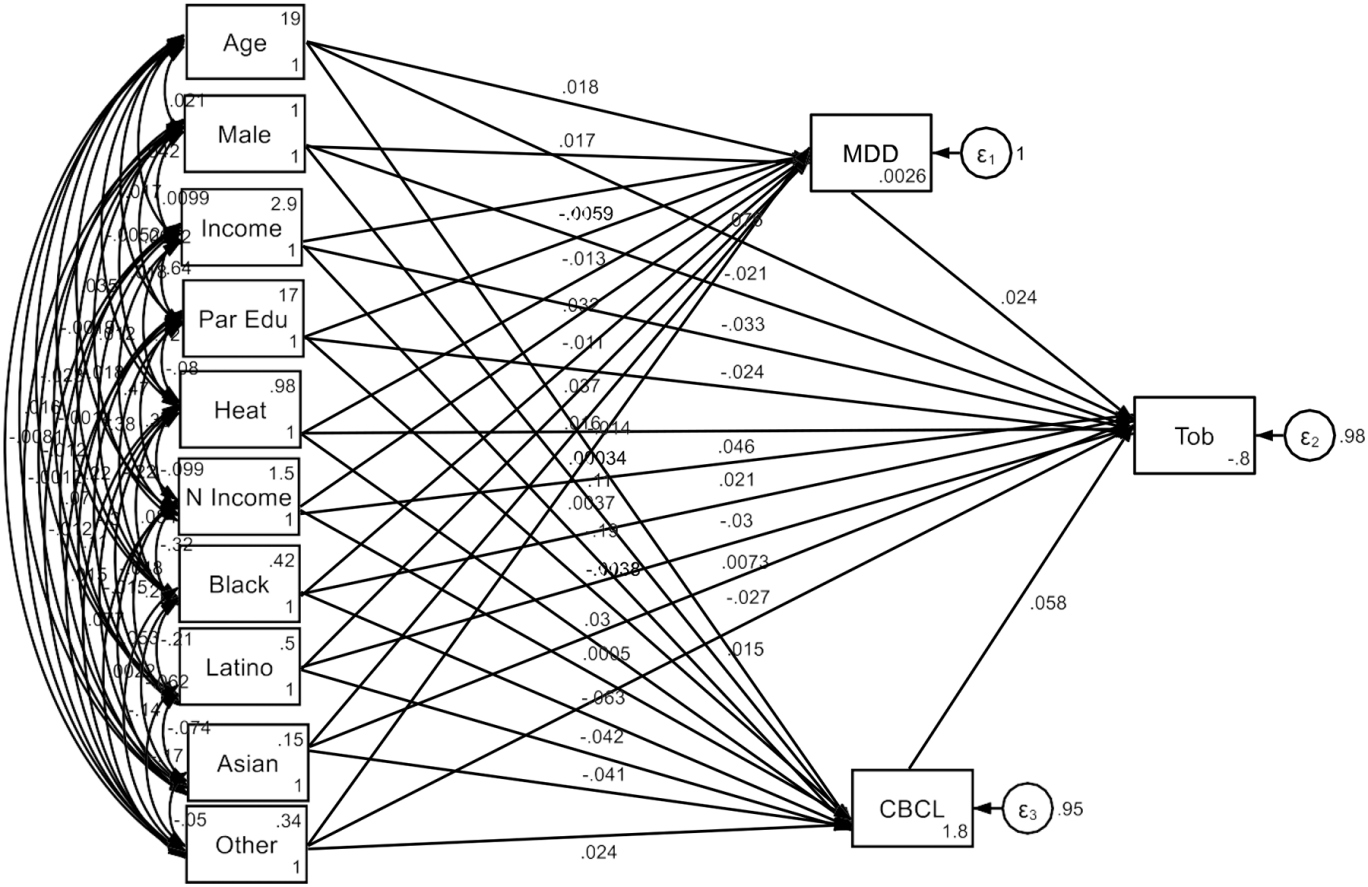
Summary of SEM

**Table 1.** Summary of the Structural Equation Model (SEM).

Independent Variable		Dependent Variable	B	SE	95%	CI	p
Age	→	MDD	0.018	0.010	−0.003	0.038	0.088
Male	→	MDD	0.017	0.010	−0.004	0.037	0.107
Family Income	→	MDD	−0.006	0.015	−0.036	0.024	0.701
Family Education	→	MDD	−0.013	0.014	−0.041	0.014	0.340
Heat Exposure	→	MDD	0.032	0.011	0.011	0.054	0.003
Neighborhood Income	→	MDD	−0.011	0.012	−0.034	0.013	0.379
Black	→	MDD	0.037	0.012	0.013	0.061	0.003
Latino	→	MDD	0.016	0.012	−0.008	0.039	0.199
Asian	→	MDD	0.000	0.011	−0.021	0.021	0.975
Other	→	MDD	0.004	0.011	−0.018	0.025	0.733
Intercept	→	MDD	0.003	0.294	−0.574	0.579	0.993
							
MDD	→	Tobacco Use Initiation	0.024	0.011	0.003	0.046	0.027
Behavioral Problems	→	Tobacco Use Initiation	0.058	0.009	0.040	0.077	< 0.001
Age	→	Tobacco Use Initiation	0.076	0.009	0.058	0.094	< 0.001
Male	→	Tobacco Use Initiation	−0.021	0.009	−0.039	−0.003	0.024
Family Income	→	Tobacco Use Initiation	−0.033	0.014	−0.061	−0.006	0.017
Family Education	→	Tobacco Use Initiation	−0.024	0.012	−0.048	0.001	0.058
Heat Exposure	→	Tobacco Use Initiation	0.046	0.010	0.027	0.065	< 0.001
Neighborhood Income	→	Tobacco Use Initiation	0.021	0.011	0.000	0.043	0.049
Black	→	Tobacco Use Initiation	−0.030	0.011	−0.052	−0.008	0.007
Latino	→	Tobacco Use Initiation	0.007	0.011	−0.014	0.028	0.494
Asian	→	Tobacco Use Initiation	−0.027	0.009	−0.045	−0.008	0.004
Other	→	Tobacco Use Initiation	0.015	0.010	−0.004	0.034	0.124
Intercept	→	Tobacco Use Initiation	−0.797	0.261	−1.308	−0.285	0.002
							
Age	→	Behavioral Problems	−0.014	0.009	−0.032	0.004	0.121
Male	→	Behavioral Problems	0.108	0.009	0.090	0.125	< 0.001
Family Income	→	Behavioral Problems	−0.190	0.013	−0.216	−0.163	< 0.001
Family Education	→	Behavioral Problems	−0.004	0.012	−0.028	0.020	0.757
Heat Exposure	→	Behavioral Problems	0.030	0.010	0.011	0.049	0.002
Neighborhood Income	→	Behavioral Problems	0.000	0.011	−0.020	0.021	0.963
Black	→	Behavioral Problems	−0.063	0.011	−0.085	−0.042	< 0.001
Latino	→	Behavioral Problems	−0.042	0.010	−0.062	−0.021	< 0.001
Asian	→	Behavioral Problems	−0.041	0.009	−0.059	−0.023	< 0.001
Other	→	Behavioral Problems	0.024	0.009	0.006	0.043	0.010
Intercept	→	Behavioral Problems	1.783	0.255	1.283	2.283	< 0.001
